# Factors associated with Senegalese health workers’ willingness to receive mobile digital payments: cross-sectional study

**DOI:** 10.1136/bmjgh-2024-017468

**Published:** 2025-11-19

**Authors:** Amadou Ibra Diallo, Mouhamadou Faly Ba, Sarah Louart, Fatoumata Binetou Diongue, Ibrahima Gaye, Emmanuel Bonnet, Adams Diedhiou, Souleymane Ndiaye, Zahra Mboup, Valery Ridde, Adama Faye

**Affiliations:** 1 Institute of Health and Development, Cheikh Anta Diop University of Dakar, Dakar, Senegal; 2 Department of Preventive Medicine and Public Health, Cheikh Anta Diop University Faculty of Medicine Pharmacy and Dentistry, Dakar, Senegal; 3 CLERSE (Lille Centre for Sociological and Economic Research and Studies), University of Lille, Lille, France; 4 French Institute for Research on Sustainable Development, Paris, France; 5 Department of Sociology, Cheikh Anta Diop University of Dakar, Dakar, Senegal; 6 Government of Senegal Ministry of Health and Social Action, Dakar, Senegal

**Keywords:** Health services research, Public Health, Health Personnel

## Abstract

**Introduction:**

Although Senegal began digitising servants’ salaries in 2004, payments to community health workers have not yet followed suit. This study explored factors associated with health professionals’ and community health workers’ willingness to receive payments via mobile digital systems.

**Methodology:**

We carried out a cross-sectional study, from October to December 2023 among healthcare staff and community health workers using telephone surveys. At national level, one district per medical region was selected by random draw for the study. In each district, data were collected from all health workers agreeing to participate, using a questionnaire instrument derived from pre-existing conceptual frameworks for the acceptability of technological innovations in healthcare. We conducted a descriptive analysis, followed by a bivariate analysis with a 5% alpha risk and a multivariate analysis.

**Results:**

We recruited 2965 healthcare workers (of whom 70.1% were women), including community health workers (70.8%) and health professionals (29.2%). The arithmetic mean age was 42.7 years (SD 11.2 years). 98.6% of the sample had access to a smartphone and 80% had internet access. Healthcare workers reported a high willingness (88.2%) to be paid by mobile digital systems.

Factors negatively associated with this willingness included contractual professional status (adjusted OR (AOR) 0.46, 95% CI 0.23 to 0.93), having been in practice for more than 10 years (AOR 0.45, 95% CI 0.28 to 0.72), perceived difficulties in using technology (AOR 0.43, 95% CI 0.23 to 0.81) and carrying out additional administrative procedures. On the other hand, the simplification of payment processes (AOR 3.45, 95% CI 1.86 to 6.32) and positive opinion from health authorities (AOR 1.85, 95% CI 1.17 to 2.94) were positively associated with willingness.

**Conclusion:**

We found that individual, socioprofessional and contextual factors are associated with health workers’ willingness to receive full digitisation of payments. Seamless integration of these systems into existing organisational structures could strengthen worker buy-in.

**WHAT IS ALREADY KNOWN ON THIS TOPIC**

- In the Senegalese healthcare system, health workers receive payments via bank transfers of salaries and direct payment by cash.
- Currently, many Sub-Saharan African countries are transitioning towards digitisation of payments, with the use of mobile money in addition to bank transfers.
- However, few studies exist on the digitisation of payments in the health sector in Senegal and in the rest of Sub-Saharan Africa.

**WHAT THIS STUDY ADDS**

- Mobile money systems are well known and accepted by healthcare providers in this setting.
- We found that geographic, socioeconomic and individual factors emerge as determinants of its adoption, suggesting differences in needs and preferences between different categories of healthcare workers.

**HOW THIS STUDY MIGHT AFFECT RESEARCH, PRACTICE OR POLICY**

- Our findings imply it is important to design differentiated strategies, tailored to the different types of health worker contracts, to maximise the effectiveness of digital payment initiatives.
- Seamless integration of digital systems into existing organisational structures could potentially increase staff buy-in, particularly in remote areas.

## Introduction

Digital payments—transactions conducted through mobile or internet-based platforms^1^ have gained increasing prominence in low- and middle-income countries, particularly since the COVID-19 pandemic.^1^ Across Africa, many countries have initiated a transition towards digital financial systems, as reflected in regional initiatives such as the guide to the digitisation of payments recently published by the Central Bank of West African States for West African Economic and Monetary Union Member States.^2^ For instance, the Economic Community of West African States has recommended switching from cash payment to digital payment for civil servants.

In Senegal, digital transition is supported by a national strategy, ‘Senegal Digital 2025’ and a sectoral digital health plan for 2018–2023^3^ Despite this political commitment, progress remains limited: only 10% of payment flows are digitised in terms of value and 6% in terms of volume.^4^ Structural constraints persist, including limited infrastructure (ie, network deficiencies or absence in some rural areas), underdeveloped financial networks^5^ and weak banking coverage in rural areas.^6^

Sub-Saharan Africa stands out as a global leader in mobile money adoption, with 469 million registered accounts—nearly 50% of the global total. In countries such as Côte d’Ivoire, Senegal and Gabon, more than 30% of adults use mobile money services, far surpassing access to traditional bank accounts.^7^ Mobile money reaches users at levels seven times greater than automated teller machines and 20 times greater than bank branches.^8^ The World Bank has highlighted the potential of digitising routine payments, such as salaries, to advance financial inclusion^9^: among the 60 million unbanked adults receiving government cash transfers globally, two-thirds own a mobile phone.^7^

While digital payments are increasingly used in health financing, most research to date has focused on mechanisms for patient payments rather than the remuneration of health workers.^10 11^ Only a limited number of studies have explored digital payments to health workers during routine activities such as vaccination campaigns. In Nigeria, one study piloted mobile money transfers to vaccination personnel and achieved a 97% success rate in reaching beneficiaries.^12^ Another study conducted in Côte d’Ivoire, Mali, Ghana and the Democratic Republic of Congo found that 83% of surveyed workers preferred mobile payments to cash, citing speed, convenience and security.^9^ Digital payment systems may offer solutions to persistent challenges faced by healthcare workers, including delayed or incomplete payments and difficulties accessing funds. More secure and transparent payment mechanisms have the potential to improve performance, quality of services, compliance with working hours and retention.^13^ They may also help reduce informal payments from patients, which are often attributed to payment irregularities.^14^ However, such evidence remains scarce and mostly confined to campaign-based activities. Nevertheless, there are potential challenges related to the lack of digital education or the low access to smartphones in some areas. Little is known about digital payments in routine service settings or among diverse categories of health workers.

Digitalisation, including digital payments, is increasingly positioned as a key pillar of future health sector reforms in Africa.^15^ In Senegal, civil servants, including many health professionals, have received salaries through bank transfers or cheques since 2004.^2^ In contrast, other cadres such as community health workers (CHWs), non-civil servants and volunteers are still often paid in cash or via informal mobile transfers. This disparity raises concerns about equitable treatment of health workers based on employment status or geographic location.^16^

Although digitalisation is advancing in the health sector, little is known about how healthcare workers themselves perceive these changes, particularly in the context of routine service delivery. Understanding their willingness to receive digital payments, and under which form, such as mobile money or bank transfer, is critical for designing inclusive, efficient and scalable payment systems. We aimed to address this gap by exploring the willingness of healthcare workers in Senegal to be paid digitally. In a context of accelerating digital transformation, the findings offer timely insights into how digital financial tools may contribute to health workforce performance and equity.

## Method

### Study framework

Senegal is a Sudanese-Sahelian country in West Africa with a surface area of 196 722 km^2^, divided into 14 administrative regions. In 2019, Senegal’s population was estimated at 16 209 125, with 50.2% of women.^17^ Nearly a quarter of the population lives in the Dakar region.^17^ The Senegalese healthcare system is organised in a three-tier pyramid structure: central, intermediate and peripheral. In 2022, the national population coverage was 6820 per doctor, 1197 women of reproductive age per state midwife and 2937 people per state nurse or nursing assistant. The coastal regions of Dakar and Ziguinchor had the best population coverage by type of qualified health worker. In 2022, Senegal had 2197 community-based health centres, 1584 health posts and 114 health centres, 40 of which were level 2.^18^

The telephone network covers the national territory and the mobile phone penetration rate was 98% (proportion of households with at least one member who has a mobile phone) in 2019, with 99% in urban areas and 96.7% in rural areas. At national level, household internet access remains low, with an access rate (Wi-Fi and/or mobile) of 29.2% in 2019. However, there are disparities in access according to the place of residence: the urban population of Dakar has greater access to the internet (53.6%), followed by other urban centres (33.9%). In rural areas, the access rate is only 14.5%.^19^

### Type and period of study

This was a descriptive and analytical cross-sectional study of healthcare staff (state and contract) and CHWs, whether or not they provided care, using telephone surveys. It was carried out between October and December 2023.

### Study population

The study population comprised both health professionals (doctors, nurses, nursing assistants and midwives) and CHWs such as ‘Bagenu Gox’ (meaning ‘neighbourhood godmothers’ in the local Wolof language), matrons, community prevention and promotion workers (who carry out awareness-raising activities) and community care workers such as home care providers. Health professionals provide healthcare and are paid by the state or health structures (long term or temporary contracts), while CHWs include populations who support health professionals and receive financial incentives depending on their activities.

### Sampling

At national level, one health district per medical region was selected by random draw. In order to obtain contact lists for the remote interviews, a request was sent to the chief medical officers of each district for information and agreement to participate and to support the implementation of the project. We compiled various telephone directory sources from the health worker database in the 14 health districts. Based on this list, we conducted an exhaustive survey by calling all health staff and CHWs.

### Data collection (tools, method, procedure, data collected)

Data were collected using a questionnaire (online supplemental file 1) based on a pre-existing conceptual framework of the acceptability of technological innovation.^20 21^ According to this framework, the acceptability of a technological innovation depends on several factors: perceived complexity, perceived disadvantages, personal emotions, social influence, perceived advantages and compatibility.^1 22^ This theoretical foundation helped us in the construction of our data collection tools and the analysis of the empirical data.

10.1136/bmjgh-2024-017468.supp1 Supplementary data

The questionnaire was converted into electronic format using the Open Data Collect application and integrated into a digital platform with an internet and telephone component.

Data were collected remotely via telephone calls made by 12 interviewers who spoke French and local languages (Diola, Malinke, Pular, Serere and Wolof) and they were tasked to fill out the questionnaire with information collected via the phone calls. They received 3 days of training on the protocol, objectives, questionnaire, use of the tablets, as well as data entry and transmission to the servers.

Following the training, a remote pre-test was carried out on a sample of 50 individuals, mainly health staff from the Dakhao district in the Fatick regional directorate, which was not included in 14 health districts selected. This pre-test helped improve the questionnaire, translate incomprehensible words and estimate the time required to administer the questionnaire.

The study was conducted in successive rounds of calls. After calling all the individuals on the initial list, a second list was created, consisting of phone numbers to call back and those that were unreachable for any reason. If a person did not respond after five call attempts, they were considered unreachable. Interviews with healthcare staff were conducted in French, the official language. CHWs, depending on their preference and understanding, were interviewed in either French or local languages.

Interviewers were selected to account for the linguistic diversity among CHWs. In case of a misunderstanding between the respondent and the interviewer, the latter was redirected to another interviewer who understands their language, who then took over the interview. This approach, involving remote interviews by telephone and online data collection, has already been used to study the acceptability of government measures to deal with COVID-19 in Senegal.^23 24^

### Data capture and analysis

The use of tablets enabled data to be entered instantaneously, and synchronisation with an online server ensured data backup and immediate database extraction on completion of data collection. Quantitative variables were described using the mean and SD, and the median surrounded by the extremes. Qualitative variables were described in terms of absolute and relative frequencies. Logistic regression was used to identify associated factors and to understand the willingness of health workers to be paid by a mobile digital system.^20 21^ The top-down stepwise selection procedure was used to build the final model. The likelihood ratio test was used to compare the nested models. The adequacy of the model was assessed using the Hosmer-Lemeshow test.^25^

### Patient and public involvement

Individual data were collected with the permission of the National Ethics Committee and regional medical officials of the Senegalese Ministry of Health and Social Action. Participants (surveyed) were not involved in the analysis, interpretation of results or writing of the manuscript.

## Result

### Personal and socioprofessional characteristics

**Figure 1:**
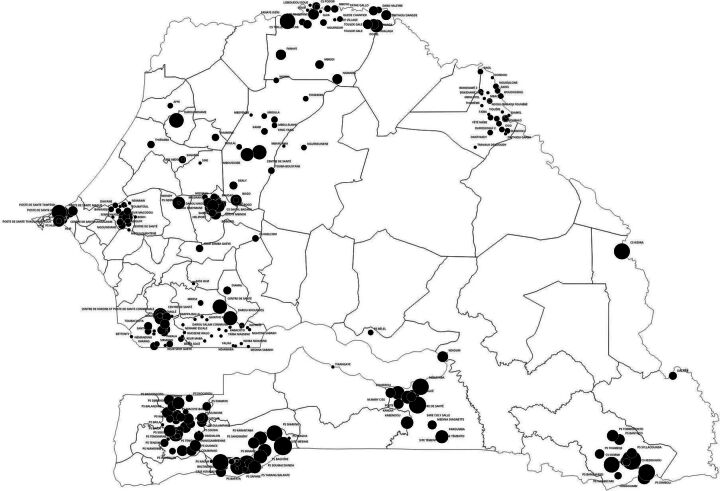
Map of respondents.

The survey was conducted among 2965 health workers, including CHWs (70.8%) and health professionals (29.2%), with 70.1% of the total sample being women. The mean age was 42.7 years, with a SD of 11.2 years. Mean age was 37.9 years (SD 6.8 years) for qualified staff and 44.6 years (SD 12.1 years) for CHWs (table 1). The most represented health districts were Touba (15.4%), Ziguinchor (14.2%), Sedhiou (13.4%) and Kolda (10.8%), spread across the central, southern and south-western parts of the country (figure 1). Just over a third (35.0%) of the workers were based in rural areas (table 2).

**Table 1:** Breakdown of health workers by personal characteristics

Variables	Terms and conditions	CHW	Healthcare professionals	Total
		(n=2099) n(%)	(n=866) n(%)	(n=2965) n(%)
District	Rufisque	115 (5.5)	26 (3.0)	141 (4.8)
	Touba	383 (18.2)	69 (8.0)	452 (15.2)
	Fatick	104 (5.0)	55 (6.4)	159 (5.4)
	Kaffrine	73 (3.5)	50 (5.8)	123 (4.1)
	Kaolack	51 (2.4)	71 (8.2)	122 (4.1)
	Kédougou	191 (9.1)	72 (8.3)	263 (8.9)
	Kolda	193 (9.2)	126 (14.5)	319 (10.8)
	Louga	76 (3.6)	46 (5.3)	122 (4.1)
	Matam	5 (0.2)	54 (6.2)	59 (2.0)
	Saint-Louis	157 (7.5)	48 (5.5)	205 (6.9)
	Sédhiou	347 (16.5)	49 (5.7)	396 (13.4)
	Tambacounda	26 (1.2)	56 (6.5)	82 (2.8)
	Thiès	65 (3.1)	36 (4.2)	101 (3.4)
	Ziguinchor	313 (14.9)	108 (12.5)	421 (14.2)
Gender	Female	1504 (71.7)	573 (66.2)	2077 (70.1)
	Male	595 (28.3)	293 (33.8)	888 (29.9)
Age	Average (SD)	44,6 (12.1)	37,9 (6.84)	42.7 (11.2)
Marital status	Single	104 (5.0)	107 (12.4)	211 (7.1)
	Divorce	35 (1.7)	21 (2.4)	56 (1.9)
	Married polygamous couple	833 (39.7)	258 (29.8)	1091 (36.8)
	Married monogamous couple	951 (45.3)	477 (55.1)	1428 (48.2)
	Widowed	176 (8.4)	3 (0.3)	179 (6.0)
Study level	Without instruction	516 (24.6)	0 (0)	516 (17.4)
	Primary	561 (26.7)	4 (0.5)	565 (19.1)
	Secondary	988 (47.1)	266 (30.7)	1254 (42.3)
	Superior	34 (1.6)	596 (68.8)	630 (21.2)

CHW, community health worker.

**Table 2:** Breakdown of health workers by socioprofessional characteristics

Variables	Terms and conditions	CHW	Healthcare professionals	Total
		(n=2099) n(%)	(n=866) n(%)	(n=2965) n(%)
Affiliation health structure	Health post	1588 (75.7)	544 (62.8)	2132 (71.9)
	Health centre	258 (12.3)	304 (35.1)	562 (19.0)
	Hospital	1 (0.0)	3 (0.3)	4 (0.1)
	Health centre	238 (11.3)	14 (1.6)	252 (8.5)
	Other (community website)	14 (0.7)	1 (0.1)	15 (0.5)
Zone	Rural	1430 (68.1)	498 (57.5)	1928 (65.0)
	Urban	669 (31.9)	368 (42.5)	1037 (35.0)
Distance to health centre	Average (SD)	32,1 (35.9)	21,3 (28.6)	29.0 (34.3)
	Median (min, max)	15.0(0, 120)	10.0(0, 195)	15.0(0, 195)
Professional status	Contract	39 (1.9)	389 (44.9)	428 (14.4)
	Volunteer	2060 (98.1)	145 (16.7)	2205 (74.4)
	Civil servant	0 (0)	332 (38.3)	332 (11.2)
Professional experience since start of career	Average (SD)	11.5 (8.92)	10.6 (5.95)	11.3 (8.18)
Professional experience current place of work	Average (SD)	10.0 (8.36)	4.87 (4.95)	8.53 (7.89)
Average monthly income (XOF)*	No income	1587 (75.6)	18 (2.1)	1605 (54.1)
	<50 000	426 (20.3)	24 (2.8)	450 (15.2)
	From 50 000 to 99 999	75 (3.6)	105 (12.1)	180 (6.1)
	From 100 000 to 1 99 999	9 (0.4)	90 (10.4)	99 (3.3)
	200 000 or more	2 (0.1)	629 (72.6)	631 (21.3)
Access to a telephone (smartphone)	No	41 (2.0)	0 (0)	41 (1.4)
	Yes	2058 (98.0)	866 (100)	2924 (98.6)
Permanent internet access	No	586 (28.0)	7 (0.8)	593 (20.0)
	Yes	1513 (72.1)	859 (99.2)	2372 (80.0)

*1 EURO = 655 XOF.

CHW, community health worker; XOF, West African CFA franc.

The majority of health workers (74.4%) were volunteers, with this proportion increasing to 98.1% among CHWs. All medical staff had a mobile phone, but only 2% of community workers did. In terms of internet access, 20% of staff did not have permanent access (table 2).

### Results according to the different dimensions of the conceptual framework

In this context, it should be noted that almost 70% of respondents lived close to banks or mobile money terminals. In addition, a majority (73.6%) were already accustomed to using digital technologies, suggesting a certain familiarity with digital tools. However, almost a third of respondents (34%) had difficulties with the direct payment system currently used in the healthcare system. Regarding the perceived advantages of digital payment, the majority of respondents viewed it as a solution that could simplify payment processes in healthcare (91.1%) and generate substantial time savings (94.4%). They also believed that the digital payment system could solve certain current payment problems (93.3%) and motivate them in their work (87.3%). In terms of perceived complexity, although the majority of respondents felt they understood how the digital payment system would work (66.1%), an even greater proportion were confident in their ability to use digital payment services (84.0%) (table 3).

**Table 3:** Description of the items in the conceptual framework for the acceptability of a technological innovation (n=2965)

Dimension	Variables	Absolute frequency (n)	Relative frequency (%)
Context	I live close to banks or mobile money terminals.	2060	69.5
	I am used to using digital technologies.	2184	73.7
	I regularly use mobile money transfer systems.	2654	89.5
	I am having a lot of trouble with the current direct payment system.	1007	34
Perceived benefits	I think digital payment could simplify the payment process for healthcare workers.	2702	91.1
	Using digital payment could save me time.	2799	94.4
	Using digital payment could make it easier for me to manage my income.	2724	91.9
	Using digital payment could prevent late payments.	2585	87.2
	I think that the digital payment system could solve some of the payment problems we are currently encountering.	2767	93.3
	Digital payment could motivate me in my work.	2587	87.3
Perceived complexity	I understand how the digital payment system could work.	1961	66.1
	Digital payment would make it easier to send and receive my income.	2811	94.8
	I think that digital payment could simplify the procedures involved compared with direct payment.	2727	92
	You are confident in your ability to use digital payment services.	2492	84
Social influence	My opinion on digital payment could be influenced by my colleagues.	686	23.1
	My opinion on digital payment could be influenced by my family or friends.	656	22.1
	My colleagues will have a rather good opinion of digital payment.	1903	64.2
	Health authorities are likely to take a positive view of digital payment.	1776	59.9
Compatibility	Digital payment would be compatible with the way payments are organised in the healthcare sector.	2368	79.9
	I have the necessary knowledge to use digital payment.	2272	76.6
	I believe that the health district has all the necessary equipment to set up a digital payment system.	1599	53.9
	I am used to using digital technologies.	2142	72.2
	The structures needed to use digital payment (banks, mobile money terminals, etc) are accessible in my locality.	2202	74.3
Perceived disadvantages	Digital payment transfer costs are unreasonable.	2569	86.6
	I think that the introduction of a digital payment system is going to cause a lot of difficulties for me.	316	10.7
	Accessing my income via digital payment would require more time and effort than via direct payment.	321	10.8
	I think that digital payment will force me to carry out more administrative procedures.	397	13.4
Personal emotions	I will feel safe sending personal information via the digital payment system.	2490	84
	I wouldn't worry about the security of financial transactions on digital payments.	2392	80.7
	I trust the digital payment system.	2665	89.9
	I want to use a digital payment system.	2671	90.1
	My feelings about digital payments are positive.	2681	90.4
Willingness to be paid by a digital system	I find it acceptable to be paid via a digital payment system.	2614	88.2

Social influence on the perception of digital payment showed that respondents felt that their colleagues (64.2%) and health authorities (59.9%) would have a good opinion of digital payment. Regarding perceived disadvantages, a large proportion of respondents (86.7%) expressed concern about the transfer costs of digital payment. Conversely, a smaller proportion (10.7%) thought that setting up a digital payment system would cause difficulties for them or require more time and effort than direct payment (10.8%). Linked to the personal emotions dimension, most respondents would feel safe sending personal information via the digital payment system (84.0%) and have no concerns about the security of financial transactions (80.7%). Healthcare workers’ willingness to use digital payment was high in 88.2% of cases (table 3).

### Factors associated with the desire to be paid through a mobile digital payment system

The study carried out on healthcare workers’ willingness to be paid through a digital system identified several associated factors (online supplemental file 2). With regard to professional status, it appears that contract employees were less in favour of using digital payment (adjusted OR (AOR) 0.46, 95% CI 0.23 to 0.93) than civil servants. Furthermore, having 10 years or more of professional experience negatively influenced the willingness to be paid through a digital system (AOR 0.45, 95% CI 0.28 to 0.72), indicating less support for digital payment among the most experienced healthcare professionals (table 4).

10.1136/bmjgh-2024-017468.supp2 Supplementary data

**Table 4:** Factors associated with willingness to be paid by a mobile digital system

Features	Willingness to use digital payment in the healthcare sector				
	Yes, N (%)	N	Adjusted OR	95% CI	P value
Status					
Civil servant	236 (71.1)	332	1	–	
Contract	332 (77.6)	428	**0.46**	**0.23 to 0.93**	0.033*
Volunteer	2 046 (92.8)	2 205	0.74	0.28 to 2.01	0.558
Professional experience					
Less than 10 years old	1 244 (90.7)	1 371	1	–	
10 years and over	1 370 (85.9)	1 594	**0.45**	**0.28 to 0.72**	0.001***
Perceived advantages					
I think digital payment could simplify the payment process for healthcare workers.					
No	93 (35.4)	263	1	–	
Yes	2 521 (93.3)	2 702	**3.45**	**1.86 to 6.32**	<0.001***
I think that the digital payment system could solve some of the payment problems we are currently encountering.					
No	118 (45.7)	258	1	–	
Yes	2 496 (92.2)	2 707	**2.36**	**1.17 to 4.63**	0.014**
Digital payment could motivate me in my work.					
No	246 (55.9)	440	1	–	
Yes	2 368 (93.8)	2 525	**2.23**	**1.29 to 3.80**	0.003**
Social influence					
Health authorities are likely to take a positive view of digital payment.					
No	947 (79.6)	1 189	1	–	
Yes	1 667 (93.9)	1 776	**1.85**	**1.17 to 2.94**	0.008**
Perceived disadvantages					
I think that the introduction of a digital payment system is going to cause a lot of difficulties for me.					
No	2 451 (92.5)	2 649	1	–	
Yes	163 (51.6)	316	**0.43**	**0.23 to 0.81**	0.009**
Accessing my income via digital payment would require more time and effort than via direct payment.					
No	2 408 (91.1)	2 644	1	–	
Yes	206 (64.2)	321	**0.38**	**0.20 to 0.73**	0.003**
I think that digital payment will force me to carry out more administrative procedures.					
No	2 291 (89.2)	2 568	1	–	
Yes	323 (81.4)	397	**0.39**	**0.18 to 0.81**	0.013*
Personal emotions					
I want to use a digital payment system.					
No	31 (10.5)	294	1	–	
Yes	2 583 (96.7)	2 671	**68.8**	**38.7 to 127**	<0.001***
My feelings about digital payments, the responses are positive.					
No	72 (25.4)	284	1	–	
Yes	2 542 (94.8)	2 681	**4.99**	**2.70 to 9.21**	<0.001***

*Significance P value < 0,05 ; ** Significance P value <0,01; ***Significance P value <0,001.

OR, Odd Ratio.

Perceived advantages of digital payment had a significant impact on healthcare workers’ willingness to be paid by a digital system. Those who believed that it would simplify payment processes were more likely to want to use it (AOR 3.45, 95% CI 1.86 to 6.32). Similarly, those who thought that digital payment would resolve current payment issues were more inclined to use it (AOR 2.36, 95% CI 1.17 to 4.63). The belief that mobile digital payment could be more motivating at work was also associated with a greater willingness to use it (AOR 2.23, 95% CI 1.29 to 3.80) (table 4).

Social influence significantly contributed to support the deployment of mobile digital payment. Individuals who expected health authorities to have a favourable opinion of digital payment were more inclined to move towards its use (AOR 1.85, 95% CI 1.17 to 2.94). Conversely, those who feared more difficulties associated with the introduction of this type of payment were less favourable (AOR 0.43, 95% CI 0.23 to 0.81). Finally, the perception that digital payment would entail more administrative procedures was also linked to reduced willingness (AOR 0.39, 95% CI 0.18 to 0.81). In terms of personal emotions, the desire to use a mobile digital payment system was associated with a higher willingness to use (AOR 68.8, 95% CI 38.7 to 127). Similarly, those with positive feelings about mobile digital payments were more in favour of this payment method (AOR 4.99, 95% CI 2.70 to 9.21) among healthcare workers (table 4).

## Discussion

We identify new insights into the willingness of health workers in Senegal to use digital payment systems for their routine professional activities. While most existing studies have focused on campaign-specific, donor-funded interventions, our research captures health workers’ perspectives in the context of day-to-day service delivery, offering a broader view of how digital payments could be integrated into routine health system operations. These findings are particularly relevant in the current wave of digital transformation in health systems across Sub-Saharan Africa.

While digital payments are not yet widely implemented, the workforce expresses strong interest and a high level of declared willingness to use them. This enthusiasm likely reflects the increasing integration of mobile financial services into everyday life in Senegal, including mobile money use for bill payments, transportation and shopping.^26^ This evolution has been driven by the diversification of mobile operators,^27^ a mobile phone penetration rate above 120% in 2023, due to the widespread use of multiple SIM cards and improved internet access.^28^

Our results revealed that contract employees and more experienced staff were significantly less willing to use digital payment systems. These findings suggest that employment stability and long-established work routines may influence openness to new digital practices. This aligns with evidence from a systematic review on technology and employment, which found that workers in temporary or precarious positions are often more hesitant to adopt new technologies due to their perception of job instability and financial confidence issues.^29^ In contexts where transaction fees are perceived as a financial burden, contract workers (who are generally paid less than civil servants) may also favour traditional cash-based payment methods that avoid additional charges.

Perceived advantages, social influence and personal emotions emerged as important determinants of health workers’ willingness to use digital payment systems. These findings are consistent with prior studies that have identified similar drivers of digital payment engagement. For instance, research has shown that tangible benefits such as convenience and accessibility are key motivators for users.^29 30^ The influence of social norms—including the approval of peers and supervisors—has also been found to play a decisive role in shaping willingness to use digital technologies in workplace settings.^31^ Furthermore, the role of affective responses and prior experiences has been highlighted in the literature, with individuals who have had positive interactions with comparable technologies more likely to express willingness to engage with new ones.^32^ A recent systematic review on mobile fintech adoption in Sub-Saharan Africa also confirmed that perceived usefulness and ease of use are key determinants of adoption.^33^ This convergence of results reinforces the importance of incorporating these behavioural and perceptual dimensions into the development of strategies to support the use of digital payment in healthcare.

In contrast, our study also revealed concerns related to security, transaction costs (around 1% of the amount transferred) that were paid by the worker, and perceived administrative burden, which may act as potential barriers to the willingness to use digital payment systems.^34^ These concerns are consistent with findings from previous research.^35^ In particular, perceived risks, such as data breaches, fraud and financial loss, as well as worries about service fees, have been widely documented as key deterrents to digital payment uptake.^34 36^

These studies also emphasise that low trust in digital platforms or institutions can further reduce engagement, especially in contexts where cash-based systems are perceived as more reliable. Moreover, uncertainty avoidance has been shown to negatively influence acceptance when digital technologies are perceived as unfamiliar or lacking transparency.^36^ Finally, we observed no significant association between professional category and willingness to use digital payment, suggesting that perceptions and attitudes may transcend occupational differences among health workers.^37^

However, stated willingness does not always lead to actual adoption. The willingness declared before the actual implementation of a programme may differ from the actual adoption once confronted with new working habits.^38^ Behavioural studies have shown that intentions may diverge from real-world behaviours.^39^ This ‘intention-behaviour gap’ underscores the need for continuous monitoring and tailored support throughout the rollout of digital payment systems in the health sector. It is therefore essential to monitor changes in perceptions as digital payment is implemented on a large scale.^40^ Future research could use longitudinal designs to follow changes in attitudes and behaviours over time, particularly after implementation begins. Such studies could help identify when and why willingness to use digital payments weakens or strengthens and how different profiles of workers respond. Indepth qualitative research would also be valuable to explore underlying concerns, perceptions of trust and contextual barriers in greater depth. A complementary qualitative study has already been conducted and will be published separately, offering further insight into the dynamics that shape acceptability.

This study points to several areas that could be considered to support the effective and equitable use of digital payment systems in the health sector. Addressing concerns related to transaction costs, through subsidised or fee-free transfers for health-related payments, may be important to ensure acceptability among lower-paid staff. Improving trust in digital platforms, particularly in rural or underserved areas, could also play a role, especially where concerns about cybersecurity, data protection and service reliability persist. Establishing a clear and inclusive regulatory framework may contribute to addressing these issues. Ensuring that digital tools are compatible with basic phones may also be relevant, given limited smartphone access among some CHWs. In addition, the role of social endorsement suggests that involving health authorities and early adopters could help foster trust and uptake. Further areas for consideration include strengthening digital literacy among health workers, particularly those in more precarious positions, and encouraging coordination between service providers through interoperable standards across mobile money platforms and banks. Exploring these dimensions, both from the demand and supply side, could contribute to the successful integration of digital payment into health systems.

### Limitations

This quantitative telephone survey could not reach all the healthcare workers from the established phone number list. Unreachable or incorrect numbers, a common limitation of telephone surveys, may have affected the representativeness of the findings. Additionally, data collected through telephone interviews may be influenced by social desirability bias: respondents might provide answers they believe are expected or socially acceptable, rather than expressing their true opinions.

A real-life follow-up study after the rollout of digital payment methods would help validate these findings.

This study opens up a number of avenues for future research. It would be relevant to extend this type of analysis to other Sub-Saharan African countries making progress in their digital transition of the healthcare sector. Future research could also focus on assessing the long-term impact of digital payment on healthcare quality and the effectiveness of public health interventions at operational level.

## Conclusion

We provide insights into the willingness of Senegalese health workers to be paid through a mobile digital system. Geographical, socioeconomic and individual factors emerge as key determinants of these dynamics. Overall, there was a high level of willingness to adopt digital mobile payment (88.2%) among those interviewed. It is imperative to devise differentiated strategies, adapted to regional realities and agent profiles, to maximise the effectiveness of mobile digital payment initiatives. It is essential to emphasise that formalising and promoting digital payment in Senegal’s healthcare sector is not just a matter of operational efficiency, but also an opportunity to catalyse economic and social development.

However, this process must be guided by an inclusive, ethical and sustainable approach, ensuring that all healthcare workers benefit equally from this digital transformation. The potential benefits in terms of efficiency, transparency and financial inclusion can position Senegal as a leader in the adoption of digital payment in healthcare in Sub-Saharan Africa.

## Data Availability

Data are available upon reasonable request. Data may be obtained from a third party and are not publicly available.
