# Supplementary material for: Factors associated with Senegalese health workers’ willingness to receive mobile digital payments: cross-sectional study
Source: BMJ Glob Health. 2025 Nov 19;10(Suppl 4):e017468. doi: 10.1136/bmjgh-2024-017468 (PMC12658536; doi:10.1136/bmjgh-2024-017468)
Supplement: Supplementary data [file bmjgh-10-Suppl_4-s001.pdf]

Supplemental File 2: Final multivariate model of Factors associated with willingness to be paid by a mobile digital system

Features	Willingness to use digital payment in the healthcare sector				
	Yes, n (%)	N	Adjusted OR	95% CI	p-value
**Agent type**					
Healthcare professionals	660 (76.2)	866	1	-	
CHW	1 954 (93.1)	2 099	2.08	0.83 – 5.07	0.112
**Region**					
Dakar	127 (90.1)	141	1	-	
Diourbel	429 (94.9)	452	1.59	0.35 – 6.40	0.53
Fatick	141 (88.7)	159	0.95	0.21 – 4.01	0.944
Kaffrine	105 (85.4)	123	0.69	0.14 – 3.14	0.637
Kaolack	87 (71.3)	122	0.36	0.08 – 1.47	0.167
Kédougou	222 (84.4)	263	0.98	0.22 – 4.03	0.979
Kolda	277 (86.8)	319	1.1	0.25 – 4.27	0.895
Louga	103 (84.4)	122	0.8	0.17 – 3.57	0.771
Matam	45 (76.3)	59	0.38	0.07 – 1.94	0.245
Saint-Louis	186 (90.7)	205	1.74	0.36 – 8.13	0.487
Sédhiou	346 (87.4)	396	0.54	0.13 – 1.86	0.36
Tambacounda	72 (87.8)	82	0.82	0.15 – 4.33	0.813
Thiès	87 (86.1)	101	0.91	0.19 – 4.15	0.91
Ziguinchor	387 (91.9)	421	3.57	0.81 – 14.4	0.083
**Gender**					
Female	1 822 (87.7)	2 077	1	-	
Male	792 (89.2)	888	1.53	0.92 – 2.58	0.106
**Status**					
Civil servant	236 (71.1)	332	1	-	
Contract	332 (77.6)	428	**0.46**	**0.23 – 0.93**	**0.033***
Volunteer	2 046 (92.8)	2 205	0.74	0.28 – 2.01	0.558
**Professional experience**					
Less than 10 years old	1 244 (90.7)	1 371	1	-	
10 years and over	1 370 (85.9)	1 594	**0.45**	**0.28 – 0.72**	**0.001*****
**Context**					
**I live close to banks or mobile money terminals**					
No	778 (86.0)	905	1	-	
Yes	1 836 (89.1)	2 060	1.57	0.96 – 2.54	0.069
**I regularly use mobile money transfer systems**					
No	242 (77.8)	311	1	-	
Yes	2 372 (89.4)	2 654	1.75	0.87 – 3.42	0.109
**I'm having a lot of problems with the current direct payment system (late payment, partial payment).**					
No	1 679 (85.8)	1 958	1	-	
Yes	935 (92.9)	1 007	1.56	0.93 – 2.68	0.097
**Perceived advantages**					
**I think digital payment could simplify the payment process for healthcare workers**					
No	93 (35.4)	263	1	-	
Yes	2 521 (93.3)	2 702	**3.45**	**1.86 – 6.32**	**<0.001**

**Features**	**Willingness to use digital payment in the healthcare sector**				
	**Yes, n (%)**	**N**	**Adjusted OR**	**95% CI**	**p-value**
					***
**Using digital payment could make it easier for me to manage my income**					
No	91 (37.8)	241	1	-	
Yes	2 523 (92.6)	2 724	1.78	0.90 – 3.47	0.093
**I think that the digital payment system could solve some of the payment problems we are currently encountering.**					
No	118 (45.7)	258	1	-	
Yes	2 496 (92.2)	2 707	**2.36**	**1.17 – 4.63**	**0.014***
**Digital payment could motivate me in my** work					
No	246 (55.9)	440	1	-	
Yes	2 368 (93.8)	2 525	**2.23**	**1.29 – 3.80**	**0.003***
**Perceived complexity**					
**I understand how the digital payment system could** work					
No	847 (84.4)	1 004	1	-	
Yes	1 767 (90.1)	1 961	0.6	0.36 – 1.01	0.056
**You are confident in your ability to use** digital **payment services**					
No	355 (75.1)	473	1	-	
Yes	2 259 (90.7)	2 492	1.77	0.96 – 3.25	0.066
**Social influence**					
**Health authorities are likely to take a positive view of** digital **payment**					
No	947 (79.6)	1 189	1	-	
Yes	1 667 (93.9)	1 776	**1.85**	**1.17 – 2.94**	**0.008***
**Compatibility**					
**I'm used to using digital technologies**					
No	734 (89.2)	823	1	-	
Yes	1 880 (87.8)	2 142	1.68	0.91 – 3.08	0.097
**Perceived disadvantages**					
**Digital payment transfer costs are reasonable**					
No	2 434 (89.2)	2 730	1	-	
Yes	180 (76.6)	235	0.58	0.29 – 1.19	0.125
**I think that the introduction of a digital payment system is going to cause a lot of difficulties for me**					
No	2 451 (92.5)	2 649	1	-	
Yes	163 (51.6)	316	**0.43**	**0.23 – 0.81**	**0.009***
**Accessing my income via digital payment would require more time and effort than via direct payment.**					
No	2 408 (91.1)	2 644	1	-	
Yes	206 (64.2)	321	**0.38**	**0.20 – 0.73**	**0.003***
**I think that digital payment will force me to carry out more administrative procedures**					
No	2 291 (89.2)	2 568	1	-	

Features	Willingness to use digital payment in the healthcare sector				
	Yes, n (%)	N	Adjusted OR	95% CI	p-value
Yes	323 (81.4)	397	**0.39**	**0.18 – 0.81**	**0.013***
**Personal emotions**					
**I won't be worried about the security of financial transactions on digital payments**					
No	384 (67.0)	573	1	-	
Yes	2 230 (93.2)	2 392	1.46	0.87 – 2.41	0.144
**I want to use a digital payment system**					
No	31 (10.5)	294	1	-	
Yes	2 583 (96.7)	2 671	**68.8**	**38.7 - 127**	**<0.001 ***
**My feelings about digital payments are positive**					
No	72 (25.4)	284	1	-	
Yes	2 542 (94.8)	2 681	**4.99**	**2.70 – 9.21**	**<0.001 ***
