# Supplementary material for: Factors associated with Senegalese health workers’ willingness to receive mobile digital payments: cross-sectional study
Source: BMJ Glob Health. 2025 Nov 19;10(Suppl 4):e017468. doi: 10.1136/bmjgh-2024-017468 (PMC12658536; doi:10.1136/bmjgh-2024-017468)
Supplement: Supplementary data [file bmjgh-10-Suppl_4-s002.pdf]

Supplemental File 1: Questionnaire on willingness to be paid by a mobile digital system among health workers

QUESTIONNAIRE NUMBER		
SURNAME AND FIRST NAME(S) OF INTERVIEWER		
**1. SOCIO-PROFESSIONAL CHARACTERISTICS**		
Q1.1 MEDICAL REGION		|____|
Q1.2 HEALTH DISTRICT	....	|____|
Q1.8 SEX	1. MALE<br>**2**. FEMALE	|____|
Q1.9 AGE (IN YEARS)		|____|
Q1.10 MARITAL STATUS	1. MARRIED MONOGAMOUS COUPLE<br>2. MARRIED POLYGAMOUS COUPLE<br>3. CELIBATE<br>4. DIVORCED<br>5. WIDOWED	|____|
Q1.11 LEVEL OF EDUCATION IN OFFICIAL LANGUAGE FRENCH	1. WITHOUT INSTRUCTION<br>2. PRIMARY<br>3. SECONDARY<br>4. HIGHER	|____|
Q1.12 IF NO EDUCATION, ARE YOU LITERATE IN THE NATIONAL LANGUAGE?	1. YES<br>2. NO	|____|
Q1.13 IF YES, IN WHICH LANGUAGES?	1. WOLOF<br>2. SERERE<br>3. PEULH<br>4. DIOLA<br>5. SONINKÉ<br>6. MANDINGUE<br>7. OTHER TO BE SPECIFIED	|____| |____| |____| ....
Q1.3 AFFILIATION STRUCTURE	1. HEALTH CENTRE<br>2. HEALTH POST<br>3. CASE<br>4. HOSPITAL<br>5. OTHER	|____|
Q1.4 NAME OF VILLAGE OR NEIGHBOURHOOD	....	|____|
Q1.6 PRACTICE AREA	1. URBAN<br>2. RURAL	
Q1.7 DISTANCE BETWEEN PLACE OF PRACTICE AND HEALTH CENTRE IN KM (Enter 0 for health centre staff)		|____|

Q1.8 SOCIO-PROFESSIONAL CATEGORY	1. PHYSICIAN<br>2. PHARMACIST<br>3. DENTAL SURGEON	|____|

Diallo AI, *et al. BMJ Glob Health* 2025; 10:e017468. doi: 10.1136/bmjgh-2024-017468

	4. NURSE<br>5. NURSING ASSISTANT<br>6. MIDWIFE<br>7. COMMUNITY HEALTH WORKER (ASC)<br>8. OTHER	
Q1.9 IF ASC, WHAT TYPES?	1. COMMUNITY HEALTH WORKER (CARE, E.G. DSDOM)<br>2. COMMUNITY HEALTH WORKER (PREVENTION/PROMOTION)<br>3. MATRONE<br>4. BAJENU GOX<br>5. OTHER	|___|
Q1.10 WHAT IS YOUR PROFESSIONAL STATUS?	1. OFFICIAL (CIVIL SERVANT)<br>2. CONTRACTUAL<br>3. VOLUNTEER	|___|
Q1.11 PROFESSIONAL EXPERIENCE SINCE THE BEGINNING OF YOUR CAREER (IN YEARS)		|___||__|
Q1.12 BIS PROFESSIONAL EXPERIENCE IN YOUR CURRENT PLACE OF WORK (IN YEARS)		|___||__|
Q1.13 USUAL MONTHLY INCOME IN FCFA?	1. NO INCOME<br>2. LESS THAN 50 000<br>3. FROM 50,000 TO 99,999<br>4. 100,000 TO 199,999<br>5. 200,000 OR MORE	|___|
Q1.14 DO YOU HAVE A MOBILE PHONE?	1. YES<br>2. NO	|___|
Q1.15 HOW DO YOU ACCESS YOUR SMARTPHONE?	1. I HAVE A SMARTPHONE<br>2. AT HOME (FAMILY MEMBER)<br>3. NEIGHBOUR / FRIEND<br>4. OTHER PLEASE SPECIFY	|___|
Q1.16 DO YOU HAVE ACCESS TO THE INTERNET?	1. YES<br>2. NO	|___|
Q1.17 HOW DO YOU ACCESS THE INTERNET?	1. HOME WIFI PAID FOR BY MYSELF<br>2. WIFI AT HOME PAID FOR BY SOMEONE OTHER THAN MYSELF<br>3. WIFI OUTSIDE THE HOME (NEIGHBOUR/FRIEND)<br>4. PURCHASE OF MOBILE DATA (PASS)<br>5. MOBILE DATA SHARING<br>6. INTERNET IN A PUBLIC PLACE<br>7. OTHER TO BE SPECIFIED	|___|
**2. DIGITAL PAYMENT (MOBILE MONEY)**		
**Context**		
I LIVE CLOSE TO BANKS OR MOBILE MONEY TERMINALS	5 COMPLETELY AGREE<br>4- AGREE<br>3-DON'T AGREE OR DISAGREE<br>2-DO NOT AGREE<br>1-DO NOT AGREE AT ALL	|___|

I'M USED TO USING DIGITAL TECHNOLOGIES	5-STRONGLY AGREE<br>4- AGREE<br>3-NEITHER AGREE NOR DISAGREE<br>2-DO NOT AGREE<br>1-DO NOT AGREE AT ALL	|____|
I REGULARLY USE MOBILE MONEY TRANSFER SYSTEMS	5-STRONGLY AGREE<br>4- AGREE<br>3-NEITHER AGREE NOR DISAGREE<br>2-DO NOT AGREE<br>1-DO NOT AGREE AT ALL	|____|
I'M HAVING A LOT OF TROUBLE WITH THE CURRENT DIRECT PAYMENT SYSTEM	5-STRONGLY AGREE<br>4- AGREE<br>3-DON'T AGREE OR DISAGREE<br>2-DO NOT AGREE<br>1-DO NOT AGREE AT ALL	|____|
**Perceived advantages**		
I THINK DIGITAL PAYMENT COULD SIMPLIFY THE PAYMENT PROCESS FOR HEALTHCARE WORKERS	5-STRONGLY AGREE<br>4- AGREE<br>3-DON'T AGREE OR DISAGREE<br>2-DO NOT AGREE<br>1-NOT AT ALL AGREE	|____|
USING DIGITAL PAYMENT COULD SAVE ME TIME	5-STRONGLY AGREE<br>4- AGREE<br>3-NEITHER AGREE NOR DISAGREE<br>2-DO NOT AGREE<br>1-DO NOT AGREE AT ALL	|____|
USING DIGITAL PAYMENT COULD MAKE IT EASIER FOR ME TO MANAGE MY INCOME	5-STRONGLY AGREE<br>4- AGREE<br>3-NEITHER AGREE NOR DISAGREE<br>2-DO NOT AGREE<br>1-DO NOT AGREE AT ALL	|____|
USING DIGITAL PAYMENT COULD PREVENT LATE PAYMENTS	5-STRONGLY AGREE<br>4- AGREE<br>3-DON'T AGREE OR DISAGREE<br>2-DO NOT AGREE<br>1-DO NOT AGREE AT ALL	|____|
I THINK THAT THE DIGITAL PAYMENT SYSTEM COULD SOLVE SOME OF THE PAYMENT PROBLEMS WE ARE CURRENTLY ENCOUNTERING.	5-STRONGLY AGREE<br>4- AGREE<br>3-DON'T AGREE OR DISAGREE<br>2-DO NOT AGREE<br>1-NOT AT ALL AGREE<br>6-NO PROBLEMS ENCOUNTERED	|____|
DIGITAL PAYMENT COULD MOTIVATE ME IN MY WORK	5-STRONGLY AGREE<br>4- AGREE<br>3-DON'T AGREE OR DISAGREE<br>2-DO NOT AGREE<br>1-DO NOT AGREE AT ALL<br>6-NO PROBLEMS ENCOUNTERED	|____|

**Perceived complexity**		
I UNDERSTAND HOW THE DIGITAL PAYMENT SYSTEM COULD WORK	5-STRONGLY AGREE<br>4- AGREE<br>3-DON'T AGREE OR DISAGREE<br>2-DO NOT AGREE<br>1-DO NOT AGREE AT ALL	|___|
DIGITAL PAYMENT WOULD MAKE IT EASIER TO SEND AND RECEIVE MY INCOME	5-STRONGLY AGREE<br>4- AGREE<br>3-DON'T AGREE OR DISAGREE<br>2-DO NOT AGREE<br>1-DO NOT AGREE AT ALL	|___|
I THINK THAT DIGITAL PAYMENT COULD SIMPLIFY THE PROCEDURES INVOLVED COMPARED WITH DIRECT PAYMENT.	5-STRONGLY AGREE<br>4- AGREE<br>3-DON'T AGREE OR DISAGREE<br>2-DO NOT AGREE<br>1-DO NOT AGREE AT ALL	|___|
YOU ARE CONFIDENT IN YOUR ABILITY TO USE DIGITAL PAYMENT SERVICES	5-STRONGLY AGREE<br>4- AGREE<br>3-NEITHER AGREE NOR DISAGREE<br>2-DO NOT AGREE<br>1-DO NOT AGREE AT ALL	|___|
**Social influence**		
MY OPINION ON DIGITAL PAYMENT COULD BE INFLUENCED BY MY COLLEAGUES	5-STRONGLY AGREE<br>4- AGREE<br>3-DON'T AGREE OR DISAGREE<br>2-DO NOT AGREE<br>1-DO NOT AGREE AT ALL	|___|
MY OPINION ON DIGITAL PAYMENT COULD BE INFLUENCED BY MY FAMILY OR FRIENDS	5-STRONGLY AGREE<br>4- AGREE<br>3-DON'T AGREE OR DISAGREE<br>2-DO NOT AGREE<br>1-DO NOT AGREE AT ALL	|___|
MY COLLEAGUES WILL HAVE A RATHER GOOD OPINION OF DIGITAL PAYMENT	5-STRONGLY AGREE<br>4- AGREE<br>3-NEITHER AGREE NOR DISAGREE<br>2-DO NOT AGREE<br>1-NOT AT ALL AGREE	|___|
HEALTH AUTHORITIES ARE LIKELY TO TAKE A POSITIVE VIEW OF DIGITAL PAYMENT	5-STRONGLY AGREE<br>4- AGREE<br>3-NEITHER AGREE NOR DISAGREE<br>2-DO NOT AGREE<br>1-NOT AT ALL AGREE	|___|
**Compatibility**		
DIGITAL PAYMENT WOULD BE COMPATIBLE WITH THE WAY PAYMENTS ARE ORGANISED IN THE HEALTHCARE SECTOR	5-FULLY AGREE<br>4- AGREE<br>3-DON'T AGREE OR DISAGREE<br>2-DISAGREE<br>1-DO NOT AGREE AT ALL	|___|

I HAVE THE NECESSARY KNOWLEDGE TO USE DIGITAL PAYMENT,	5-STRONGLY AGREE<br>4- AGREE<br>3-DON'T AGREE OR DISAGREE<br>2-DO NOT AGREE<br>1-DO NOT AGREE AT ALL	|___|
I BELIEVE THAT THE HEALTH DISTRICT HAS ALL THE NECESSARY EQUIPMENT TO SET UP A DIGITAL PAYMENT SYSTEM.	5-STRONGLY AGREE<br>4- AGREE<br>3-DON'T AGREE OR DISAGREE<br>2-DO NOT AGREE<br>1-DO NOT AGREE AT ALL	|___|
I'M USED TO USING DIGITAL TECHNOLOGIES	5-STRONGLY AGREE<br>4- AGREE<br>3-NEITHER AGREE NOR DISAGREE<br>2-DO NOT AGREE<br>1-DO NOT AGREE AT ALL	|___|
THE STRUCTURES NEEDED TO USE DIGITAL PAYMENT (BANKS, MOBILE MONEY TERMINALS, ETC.) ARE ACCESSIBLE IN MY LOCALITY.	5-STATELY AGREE<br>4- AGREE<br>3-DON'T AGREE OR DISAGREE<br>2-DO NOT AGREE<br>1-NOT AT ALL AGREE	|___|
**Perceived difficulties/disadvantages**		
DIGITAL PAYMENT TRANSFER COSTS ARE UNREASONABLE	5-STRONGLY AGREE<br>4- AGREE<br>3-DON'T AGREE OR DISAGREE<br>2-DO NOT AGREE<br>1-NOT AT ALL AGREE	|___|
I THINK THAT THE INTRODUCTION OF A DIGITAL PAYMENT SYSTEM IS GOING TO CAUSE A LOT OF DIFFICULTIES FOR ME	5-STRONGLY AGREE<br>4- AGREE<br>3-DON'T AGREE OR DISAGREE<br>2-DO NOT AGREE<br>1-DO NOT AGREE AT ALL	|___|
ACCESSING MY INCOME VIA DIGITAL PAYMENT WOULD REQUIRE MORE TIME AND EFFORT THAN VIA DIRECT PAYMENT.	5-STRONGLY AGREE<br>4- AGREE<br>3-DON'T AGREE OR DISAGREE<br>2-DO NOT AGREE<br>1-DO NOT AGREE AT ALL	|___|
I THINK THAT DIGITAL PAYMENT WILL FORCE ME TO CARRY OUT MORE ADMINISTRATIVE PROCEDURES	5-STRONGLY AGREE<br>4- AGREE<br>3-DON'T AGREE OR DISAGREE<br>2-DO NOT AGREE<br>1-DO NOT AGREE AT ALL	|___|
**Personal affections, emotions**		
I WILL FEEL SAFE SENDING PERSONAL INFORMATION VIA THE DIGITAL PAYMENT SYSTEM	5-STRONGLY AGREE<br>4- AGREE<br>3-NEITHER AGREE NOR DISAGREE<br>2-DO NOT AGREE<br>1-DO NOT AGREE AT ALL	|___|
I WOULDN'T WORRY ABOUT THE SECURITY	5-STRONGLY AGREE	|___|

<table>
<tr><td>OF FINANCIAL TRANSACTIONS ON DIGITAL PAYMENTS,</td><td>4- AGREE
3-DON'T AGREE OR DISAGREE
2-DO NOT AGREE
1-DO NOT AGREE AT ALL</td><td></td></tr>
<tr><td>I TRUST THE DIGITAL PAYMENT SYSTEM</td><td>5-STRONGLY AGREE
4- AGREE
3-NEITHER AGREE NOR DISAGREE
2-DO NOT AGREE
1-DO NOT AGREE AT ALL</td><td>|____|</td></tr>
<tr><td>I WANT TO USE A DIGITAL PAYMENT SYSTEM</td><td>5-STRONGLY AGREE
4- AGREE
3-NEITHER AGREE NOR DISAGREE
2-DO NOT AGREE
1-DO NOT AGREE AT ALL</td><td>|____|</td></tr>
<tr><td>MY FEELINGS ABOUT DIGITAL PAYMENTS ARE POSITIVE</td><td>5-FULLY AGREE
4- AGREE
3-DON'T AGREE OR DISAGREE
2-DO NOT AGREE
1-DO NOT AGREE AT ALL</td><td>|____|</td></tr>
<tr><td colspan="3">ACCEPTABILITY</td></tr>
<tr><td>I find it acceptable to be paid via a digital payment system</td><td>5-FULLY AGREE
4- AGREE
3-DON'T AGREE OR DISAGREE
2-DO NOT AGREE
1-DO NOT AGREE AT ALL</td><td>|____|</td></tr>
<tr><td>2. IF 1 OR 2, WHY?</td><td>1- FASTER
2- MORE SAFE
3- ANONYMOUS
4- TRACEABLE
5- EASIER
6- SAVES TIME IN ROUTINE ACTIVITIES
7- IMPROVES PERFORMANCE INDIRECTLY IN ROUTINE ACTIVITIES
8- OTHER, PLEASE SPECIFY</td><td>|____|
|____|
|____|
|____|
|____|
|____|
|____|</td></tr>
<tr><td>IF 1 OR 2, THROUGH WHICH HEALTH PROGRAMMES?</td><td>1. EPI CAMPAIGN
2. MILDA CAMPAIGN
3. DMM CAMPAIGN
4. ROUTINE ACTIVITIES
5. NONE
6. DON'T KNOW
7. OTHER (S) PLEASE SPECIFY</td><td>|____|
|____|
|____|
|____|
|____|
|____|
.......</td></tr>
<tr><td>BY WHICH MOBILE MONEY OPERATORS WOULD YOU LIKE TO RECEIVE INCENTIVES?</td><td>1. ORANGE MONEY
2. WAVE
3. FREE MONEY
4. OTHER (S) PLEASE SPECIFY</td><td>|____|
|____|
|____|
............</td></tr>
</table>

IF 4 OR 5, WHY?	1-BECAUSE OF TRANSFER/WITHDRAWAL FEES<br>2-SERVICE NOT AVAILABLE IN THE AREA<br>3-SOME AMOUNTS DIFFICULT TO WITHDRAW<br>4-BECAUSE OF LACK OF TELEPHONE/INTERNET NETWORK<br>5-I SPEND MORE WITH DIGITAL PAYMENT<br>6-BECAUSE OF TRANSFER/PAYMENT ERRORS	|____| |____| |____| |____| |____| |____|
